# Machine learning–derived genetic risk scores identify IL21 as a predictor of response to omalizumab and dupilumab in asthma

**DOI:** 10.3389/falgy.2025.1670783

**Published:** 2025-10-01

**Authors:** Ayobami Akenroye, Chengyue Zhang, Tanawin Nopsopon, Sean Kalra, Scott T. Weiss, Matthew R. Moll

**Affiliations:** 1 Division of Allergy and Clinical Immunology, Department of Medicine, Brigham and Women’s Hospital and Harvard Medical School, Boston, MA, United States; 2 Channing Division of Network Medicine, Department of Medicine, Brigham and Women’s Hospital and Harvard Medical School, Boston, MA, United States; 3 Division of Pulmonary and Critical Care Medicine, Brigham and Women’s Hospital and Harvard Medical School, Boston, MA, United States; 4 Section on Pulmonary, Allergy, Critical Care, and Sleep Medicine, Department of Veterans Affairs Boston Healthcare System, West Roxbury, MA, United States

**Keywords:** asthma, monoclonal antibodies, IL-21, polygenic risk score, omalizumab, dupilumab, mepolizumab, biomarker

## Abstract

**Rationale:**

Genetic risk scores (GRS) of Th1/2/17-related loci may be associated with response to biologics. We leveraged previously published machine learning-derived GRSs associated with plasma proteins from the INTERVAL/UK-Biobank study.

**Methods:**

We assessed 42 Th1/2/17-related GRSs and SNPs for association with response (≥50% reduction in exacerbations) to biologics in 172 White patients with moderate-to-severe asthma in the Mass General Brigham Biobank (MGBB: 92 omalizumab, 38 mepolizumab, 42 dupilumab). Replication was sought in 243 individuals in the All of Us (AoU) cohort (111 omalizumab, 58 mepolizumab, 74 dupilumab). Models adjusted for age, sex, BMI, baseline exacerbations, and principal components 1–10. AUROC was used to evaluate top predictors; type I error was assessed using random GRS sets (target FDR ≤20%).

**Results:**

Females comprised a large proportion; mean BMI was 28–35 kg/m^2^. *IL21* GRS was associated with omalizumab response in MGBB (OR: 1.7, 95% CI: 1.03–2.87) with similar direction in AoU (1.5, 0.91–2.45). *IL21* also predicted dupilumab response in MGBB (2.4, 1.05–5.44) but in the opposite direction in AoU (0.57, 0.31–1.06). *IL21* replicated as a predictor of omalizumab [AUROC, 95% CI: MGBB 0.62 (0.50–0.74), AoU: 0.71 (0.61–0.81)] and dupilumab [AUROC, 95% CI, MGBB 0.76 (0.58–0.95), AoU: 0.75 (0.64–0.86)]. Adding *IL5RA* (omalizumab) or *CCL17* (dupilumab) modestly improved AUROC but not significantly. No GRS predicted mepolizumab response.

**Conclusions:**

Using ML-based GRS applied to an independent cohort of asthma patients, we found that IL-21-related GRSs were predictors of response to omalizumab and dupilumab.

## Introduction

There are six monoclonal antibodies currently approved for the treatment of asthma. These therapies target various cytokines and pathways, including immunoglobulin E (IgE), interleukin-5 (IL-5) and its receptor, IL-4/IL-13, and thymic stromal lymphopoietin (TSLP) (1). While these therapies have indeed revolutionized the care of asthma, there are opportunities to optimize their use. There is a high overlap in eligibility for these therapies making therapy selection challenging and many patients who meet eligibility for these therapies demonstrate suboptimal response (2). While blood eosinophil count (BEC) helps identify patients most likely to benefit from biologic therapy, a significant subset of patients experience little to no response, highlighting a critical challenge given the high cost of these treatments (1). Thus, identifying additional biomarkers predictive of response to respiratory biologics is important.

There is ample evidence that genetic polymorphisms contribute to the development of both asthma and COPD as well as to asthma endotypes and severity (3). Genetic risk scores (GRS) have been shown to improve the predictive accuracy of clinical models in predicting asthma risk and severity. In a recent study that sought to optimize and validate GRS for ten common chronic conditions, asthma was one of the top conditions with the highest predictive accuracy (4). Genetically-predicted protein levels have helped uncover mechanisms of asthma risk by leveraging the fact that alterations in levels of genetically regulated proteins are more likely to be causal rather than a consequence of disease activity or confounders (5). Our study objective was to identify whether GRS of T helper-1 (Th-1), Th2-, and Th-17-related loci may be associated with response to omalizumab (anti-IgE), mepolizumab (anti-IL5), or to dupilumab (anti-IL4Rα) in two real-world biobank-derived cohorts.

## Methods

This study leveraged previously published machine learning-derived GRS calculated from 50,000 healthy blood donors from the UK Biobank-Interval Study (available at https://www.omicspred.org/) that predicted SomaScan protein levels with *R*^2^ > 0.01 (6). We calculated the GRS associated with 42 pre-selected Th1/2/17-related loci in White participants from the Mass General Brigham Biobank (MGBB) who initiated dupilumab, mepolizumab, or omalizumab for the treatment of moderate-to-severe asthma. These included loci associated with the canonical Th2 cytokines and their receptors, various chemoattractants and ligands, such as CCL17, alarmins and alarmin receptors (IL-25 and ILRL1/ST-2), IL-17-related cytokines and receptors, other inflammatory or anti-inflammatory cytokine, such as IL-21 and interferon-gamma (IFN-γ) (Table 1). These GRS were rank normalized to facilitate statistical analysis. GRS that remained non-normal were excluded and for GRS with trimodal distributions, we extracted allele dosages (0,1,2) from the single nucleotide polymorphism (SNP) with the largest effects size in the score. Given the biological relevance of the IL-4 receptor, we also included *IL4R* SNPs reported in OMIM and the GWAS catalog (rs1805010, rs1801275, rs1803013, and rs1805015) as *a priori* variants of interest. Altogether, 22 GRS and 14 SNPs (Table 1 and Supplementary Table E1) were carried forward for final analyses.

**Table 1:** T-helper (Th)-1/2/17-related loci (*n* = 42) and 12 additional SNPs evaluated.

Included in final analyses (*n* = 22)	Excluded from final analyses (*n* = 20)	SNPs included in final analyses (*n* = 14)
CCL17.3519.3.2	IL1B.3037.62.1	rs1976391
CCL22.3508.78.3	IL4R.3055.54.2	rs4540249
CXCL1.2985.35.1	IFNA14.7180.114.3	rs4959105
IGFLR1.7244.16.3	CCL1.2770.51.2	rs62143196
IL12B.IL23A.10365.132.3	IGHE.IGK.IGL.4135.84.2	rs193150712
IL17RA.2992.59.2	IFNAR1.9183.7.3	rs71640035
IL17RB.5084.154.3	IFNGR2.9180.6.3	rs114163150
IL17RD.3376.49.2	IGF1.2952.75.2	rs7870825
IL1RAP.14048.7.3	IL10.2773.50.2	rs1801275
IL1RAP.2630.12.2	IRF4.9857.38.3	rs1805010
IL1RL1.4234.8.2	IL22.2778.10.2	rs1805015
IL1RL2.2994.71.2	IFNG.14147.50.3	rs1805013
IL2.3070.1.2	IRF6.9999.1.3	rs117439560
IL21.7124.18.3	IFNGR1.5825.49.3	rs9881048
IL25.4137.57.2	IFNA10.14128.121.3	
IL5.3741.4.3	IL17A.9170.24.3	
IL5RA.13686.2.3	LTBR.2636.10.2	
IL5RA.4491.4.2	LTA.LTB.3506.49.1	
IRF2.12801.33.3	IL6.4673.13.2	
PGD.4187.49.2		
TNFAIP6.5036.50.1		
TNFAIP8.12563.2.3		

SNP, single nucleotide polymorphism.

For outcomes, we defined exacerbations as a patient with moderate-to-severe persistent asthma having a visit with diagnostic code for an asthma exacerbation or having an ICD-code for an asthma-related event, such as wheezing or dyspnea, and a prescription for an oral corticosteroid (OCS) for 3–28 days within 7 days of the asthma-related event (Supplementary Tables E2 and E3). We defined response as a reduction in baseline exacerbations by ≥50% over the 12 months following biologic initiation. Models were adjusted for age, sex, body mass index (BMI), and exacerbations in the year prior to biologic initiation. We censored individuals at the time of switching to an alternate biologic. GRS were adjusted for ancestry using residuals from a linear model regressed on the first 10 principal components (PCs). We used all patients with available data. We validated our results in an independent external dataset, the All of Us (AoU) research program, a large-scale National Institutes of Health (NIH)-sponsored initiative that includes clinical and genomics data from a diverse group of Americans and currently has over 200,000 enrollees with genetic data (7). We limited our analysis to patients who self-identified as White as we did in the MGBB cohort given that the GRS were trained in a European ancestry population. We used a *p*-value of <0.20 to evaluate replication. We evaluated for type 1 error using two random samples of 32 GRS from all GRSs available from the INTERVAL study with a target false discovery rate of ≤20%. For replicated signals, we evaluated predictive accuracy using the area-under-the-receiver-operating-characteristic (AUROC) curve. DeLong *p*-values were used to compare model performances (AUCs), with *p*-values <0.05 considered significantly different. To assess if risk estimates are well calibrated within each cohort, we constructed calibration plots of observed vs. expected biologic responsiveness and used Hosmer–Lemeshow tests to evaluate for the evidence of miscalibration. We also constructed confusion matrices of predicted probabilities vs. observed event rates and evaluated the model performance across the cohorts. Additional details of our methods are in the Supplementary File. The MGB IRB this study (2021P003536). The AoU analyses were conducted in the AoU Researcher Workbench. Analyses were conducted using PLINK v2.0, R 4.2.0 with the MASS 7.3 package for negative binomial modeling and pROC version 1.18.5 for ROC.

## Results

**Figure 1:**
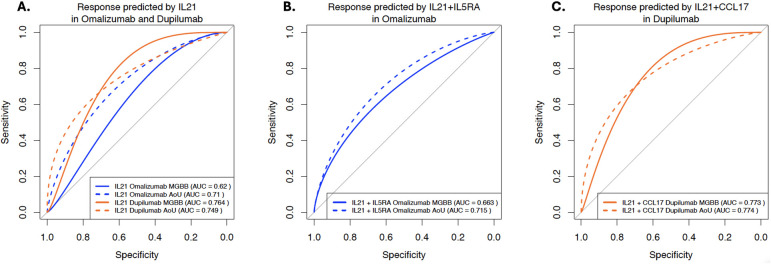
**(A–C)** Response to omalizumab and dupilumab as predicted by IL21.7124.18.3 and in combination with *IL5RA* (omalizumab) and *CCL17* (dupilumab). For **(A)** AUC and 95% CI: IL-21: Omalizumab: MGBB 0.62 (0.50–0.74), AoU: 0.71 (0.61–0.81); Dupilumab: MGBB 0.76 (0.58–0.95), AoU: 0.75 (0.64–0.86); **(B)** AUC and 95% CI: IL-21 + IL5RA: Omalizumab: MGBB 0.66 (0.55–0.78), AoU: 0.72 (0.61–0.82); **(C)** AUC and 95% CI: IL-21 + CCL17: Dupilumab: MGBB 0.77 (0.60–0.95), AoU: 0.77 (0.67–0.88).

The MGBB cohort included 172 patients: dupilumab (*n* = 42), mepolizumab (*n* = 38), and omalizumab (*n* = 92). The mean age was 60.6 for dupilumab users, 55.7 for mepolizumab, and 45.4 years for omalizumab, and mean BMI ranged from 27.7–30.4 kg/m^2^. In AoU, we identified 243 patients with moderate-to-severe asthma who initiated dupilumab (*n* = 74), mepolizumab (*n* = 58), or omalizumab (*n* = 111). Their mean ages were 60.1, 57.1, and 53.6 years respectively and mean BMI ranged from 29.3–35.2 kg/m^2^. Baseline exacerbations in MGBB were 1.2 (dupilumab), 2.7 (mepolizumab), and 1.5 (omalizumab). In AoU, they were: 2.7 (dupilumab), 6.2 (mepolizumab), and 4.5 (omalizumab) (Table 2). For omalizumab, *IL21* was most associated with response with higher levels associated with better response in both the MGBB (Odds Ratio, OR and 95% Confidence Intervals, CI: 1.72, 1.03–2.87) and AoU (OR, 95% CI: 1.50, 0.91–2.45) cohorts (Table 3). Increased allele dose of the *IL5RA* SNP were also associated with better response in both cohorts but did not reach statistical significance in either cohort. No GRS was significantly associated with mepolizumab response. For mepolizumab, some variants with *p* < 0.20 (*IGHE* and *IRF6*) had opposing effects in the cohorts. For dupilumab, *IL21* allele dose was associated with higher odds of response (OR: 2.39; 1.05–5.44) in the MGBB cohort, but with lower odds in AoU (0.57; 0.31–1.06), though crossed the null. CCL17 showed a similar trend (Table 3). Multiple variants were used in calculating the GRS for *IL21* (*n* = 157), *IL5RA* (*n* = 29), and *CCL17* (*n* = 42) (Supplementary Tables E4–E6). In the AUROC analyses, *IL21* as a predictor of response to omalizumab [AUROC and 95% CI: MGBB 0.62 (0.50–0.74), AoU: 0.71 (0.61–0.81)] and dupilumab [AUROC and 95% CI, MGBB 0.76 (0.58–0.95), AoU: 0.75 (0.64–0.86)] replicated across cohorts (Figure 1A). The AUROC increased for both biologics in both cohorts when adding *IL5RA* to *IL21* for omalizumab and *CCL17* to *IL21* for dupilumab (Figures 1B,C). However, these were not significantly different with DeLong *p*-values all >0.05 (Supplementary Table E7). A sensitivity analysis using randomly selected GRS found a type 1 error rate of 0.24. Calibration plots showed no significant miscalibration (Hosmer-Lemeshow *p*-value >0.05) (Supplementary Table E8) and confusion matrix revealed accuracy of 0.56 for *IL21* (omalizumab) in MGBB and 0.71 in AoU and 0.76 in MGBB for dupilumab with 0.66 in AoU (Supplementary Table E9). The sensitivity for *IL21* for omalizumab was low in both cohorts (0.50 MGBB; 0.35 AoU) with positive predictive value (PPV) of 0.55 and 0.65 respectively. For dupilumab, the sensitivity was high in both cohorts (0.90 MGBB; 0.76 AoU) with PPV of 0.69 and 0.67 respectively.

**Table 2:** Baseline characteristics of Mass General Brigham Biobank (MGBB) and All of US (AoU) cohort.

MGBB	Dupilumab	Mepolizumab	Omalizumab
*n*	42	38	92
Age [mean (SD)]	60.6 (18.3)	55.7 (14.8)	45.4 (15.8)
Female = 1 (%)	27 (64.3)	27 (71.1)	73 (79.3)
White Non-Hispanic, *n* (%)	42 (100.0)	38 (100.0)	92 (100.0)
Body mass index, BMI [mean (SD)]	28.1 (6.6)	27.7 (6.8)	30.4 (8.7)
Smoking (%)			
CurrentSmoker	0 (0.0)	0 (0.0)	6 (6.5)
FormerSmoker	7 (16.7)	11 (28.9)	10 (10.9)
NeverSmoker	26 (61.9)	15 (39.5)	45 (48.9)
Unknown	9 (21.4)	12 (31.6)	31 (33.7)
Baseline annual exacerbation rate (mean, SD)	1.2 (1.2)	2.7 (2.6)	1.5 (1.9)
AoU	Dupilumab	Mepolizumab	Omalizumab
*n*	74	58	111
Age [mean (SD)]	60.1 (18.0)	57.1 (13.9)	53.6 (15.7)
Female = 1 (%)	45 (60.8)	47 (82.5)	81 (73.6)
White non-Hispanic, *n* (%)	74 (100.0)	58 (100.0)	111 (100.0)
Body mass index, BMI [mean (SD)]	29.3 (7.2)	35.2 (9.0)	31.5 (8.9)
Smoking (%)			
CurrentSmoker	12 (17.1)	9 (15.5)	19 (17.3)
FormerSmoker	16 (22.9)	16 (27.6)	24 (21.8)
NeverSmoker	42 (60.0)	33 (56.9)	67 (60.9)
Unknown	0 (0.0)	0 (0.0)	0 (0.0)
Baseline annual exacerbation rate (mean, SD)	2.7 (4.1)	6.2 (4.5)	4.5 (5.4)

SD, standard deviation.

**Table 3:** Genetic risk scores associated with response that replicated in both cohorts (mass general brigham biobank-MGBB and All of US research-AoU).

Predictor	MGB Biobank (MGBB)		All of us (AoU)	
	Odds ratio (95% CI)	*P*-value*	Odds ratio (95% CI)	*P*-value*
OMALIZUMAB	Responder: 48 Non-responder: 44		Responder: 37 Non-responder: 74	
Same direction of effect				
IL21.7124.18.3	1.72 (1.03–2.87)	0.04	1.50 (0.91–2.45)	0.11
IL5RA.4491.4.2	1.46 (0.94–2.28)	0.09	1.44 (0.91–2.27)	0.12
Top associations, replicated but opposing direction of effect				
N/A				
MEPOLIZUMAB	Responder: 17 Non-responder: 21		Responder: 10 Non-responder: 48	
Same direction of effect				
N/A				
Top associations, replicated but opposing direction of effect				
IGHE.IGK.IGL.4135.84.2_snp	72.08 (0.71–7,309.55)	0.07	0.19 (0.03–1.35)	0.10
IRF6.9999.1.3_snp	6.42 (0.60–69.01)	0.13	0.08 (0.00–2.79)	0.16
DUPILUMAB	Responder: 30 Non-responder: 12		Responder: 41 Non-responder: 33	
Same direction of effect				
N/A				
Top associations, replicated but opposing direction of effect				
IL21.7124.18.3	2.39 (1.05–5.44)	0.04	0.57 (0.31–1.06)	0.08
CCL17.3519.3.2	2.29 (0.75–7.00)	0.15	0.54 (0.30–0.97)	0.04

* Including associations with *p*-value <0.20.

The GRS effects are reported per standard deviation, and the SNP effects are per allele dose.

## Discussion

In this study, we found associations between Th1/2/17-related GRS and response to biologics used in the treatment of asthma. For both omalizumab and dupilumab, an *IL21*-related GRS significantly differentiated responders from nonresponders and was replicated in the independent All of Us cohort, though there was an inverse association with dupilumab across cohorts.

Similar to a recent study using genetically predicted protein levels to uncover mechanisms underlying asthma (5), we leveraged the causal relationship between genetics and proteins, along with increasingly rich biobank data, to identify protein biomarkers for responsiveness to several biologics. Our findings are consistent with evidence that there is an interplay between Th-1, Th2-, and Th-17 pathways in asthma and in treatment response (8). We also add to the evidence that the addition of genetic risk scores to clinical variables can enhance clinical models predicting risk of obstructive lung diseases and severity across diverse populations (9).

*IL21* predicted response to dupilumab and omalizumab, which are both effective in allergic asthma. In murine models, IL-21 has been shown to modulate allergic inflammation and IgE production (10). IL-21 increased ILC2 numbers in both the airways and bronchoalveolar fluid (BAL) in mouse models, and an anti-IL21 antibody obliterates house dust mite-induced airway inflammation in murine models reducing IgE and eosinophilia. Anti-IL21 antibody also worked synergistically with anti-IL9 antibody in reducing both Th2 and ILC2 cells as well as reducing MUC5AC and bronchial hyperreactivity. In the same study, patients with allergic asthma had higher levels of IL-21 and IL-9 levels in their BAL and increased *IL21R* transcripts from their endobronchial brushings when compared to allergic controls (10). Identifying a shared biomarker of response across biologics would be clinically valuable but requires confirmation in prospective studies and also at the protein level.

While we sought to validate our findings in an independent cohort, our findings should be interpreted cautiously. First, we limited to patients who self-identified as White as a crude proxy for genetic ancestry and adjusted for the principal components of genetic ancestry. Cross-ancestry genetic prediction is a major issue in genetics, and one that we are not able to address in the current study; indeed, larger multi-ancestry cohorts and improved analytical methods are needed to ensure equitable use and representation of genetic prediction tools. Secondly, we did not adjust for multiple testing given our small sample size. Instead, using a *p* < 0.20 threshold and randomly selected unrelated GRS in sensitivity analyses, we observed type 1 error rates consistent with this threshold. Our relatively small sample size may have limited our power to detect associations, particularly in the mepolizumab group. Thirdly, we were not able to ascertain medication use or adherence or discontinuations. Lastly, there was some inverse directionality of the *IL21* GRS effects on dupilumab across cohorts. However, there are several cohort differences that may explain this phenomenon. None of the 42 dupilumab users in MGBB were current smokers and only 16.7% were former smokers. By contrast, in AoU, 40% of patients (Current-17.1%; former- 22.9%) were current/former smokers. Additionally, AoU had a higher burden of exacerbations (2.7 vs. 1.2). These cohort differences might explain the inverse effects across cohorts.

Nonetheless, these findings highlight the potential utility of GRS in predicting response to biologics in asthma and warrant further studies given the urgent need for accurate response biomarkers to these costly therapies that are rapidly increasing in number.

## Data Availability

The data analyzed in this study is subject to the following licenses/restrictions: The GRS used for this were extracted from the publicly available data from the INTERVAL study. Details on how to access these have been included in the manuscript/supplement. For MGBB data, a data use agreement needs to be set up with Mass General Brigham. Interested collaborators can also reach out to the authors for guidance, as appropriate. All of Us data is as managed by the All of Us research program. Requests to access these datasets should be directed to the corresponding authors, aakenroye@mgb.org or remol@channing.harvard.edu.
