# Supplementary material for: Machine learning–derived genetic risk scores identify IL21 as a predictor of response to omalizumab and dupilumab in asthma
Source: Front Allergy. 2025 Oct 1;6:1670783. doi: 10.3389/falgy.2025.1670783 (PMC12521206; doi:10.3389/falgy.2025.1670783)
Supplement: Supplementary file 1 [file Table1.docx]

| **Table E1: Included SNPs: rsID and Variant Information** | | |
| --- | --- | --- |
| Score | Variant_1 | rsID |
| IFNG.14147.50.3 | 2:233757337:A:G | rs1976391 |
| IFNGR2.9180.6.3 | 6:170177225:G:T | rs4540249 |
| IGHE.IGK.IGL.4135.84.2 | 6:32615369:C:T | rs4959105 |
| IL10.2773.50.2 | 19:53817382:A:G | rs62143196 |
| IL13RA1.2633.52.2_15_68544807 | 15:68544807:C:T | rs193150712 |
| IL13RA1.2633.52.2_4_186239907 | 4:186239907:T:G | rs71640035 |
| IL17A.9170.24.3 | 5:24337093:A:G | rs114163150 |
| IL22.2778.10.2 | 9:12201384:T:C | rs7870825 |
| IL4R.3055.54.2_16_27363079 | 16:27363079:A:G | rs1801275 |
| IL4R.3055.54.2_16_27344882 | 16:27344882:A:G | rs1805010 |
| IL4R.3055.54.2_16_27362859 | 16:27362859:T:C | rs1805015 |
| IL4R.3055.54.2_16_27362659 | 16:27362659:C:T | rs1805013 |
| IL6.4673.13.2 | 12:33925202:A:C | rs117439560 |
| IRF6.9999.1.3 | 3:165788464:A:C | rs9881048 |
|  |  |  |

| **Table E2: ICD codes used in defining exacerbations** | | |
| --- | --- | --- |
| Code | ICD_Version | Description |
| Diagnostic codes |  |  |
| 493 | ICD-9 | Asthma |
| 786.07 | ICD-9 | Wheezing |
| 519.11 | ICD-9 | Acute respiratory failure |
| 786.2 | ICD-9 | Cough |
| 786.05 | ICD-9 | Shortness of breath (dyspnea) |
| 786.06 | ICD-9 | Tachypnea (rapid breathing) |
| 786.01 | ICD-9 | Stridor |
| 786.09 | ICD-9 | Other respiratory abnormalities |
| J45. | ICD-10 | Asthma |
| R06.2 | ICD-10 | Wheezing |
| J98.01 | ICD-10 | Acute bronchospasm |
| R05.1 | ICD-10 | Acute cough |
| R05.8 | ICD-10 | Other specified cough |
| R05.9 | ICD-10 | Cough, unspecified |
| R06.02 | ICD-10 | Shortness of breath (dyspnea) |
| R06.82 | ICD-10 | Tachypnea, not elsewhere classified |
| R06.4 | ICD-10 | Hyperventilation |
| R06.00 | ICD-10 | Dyspnea, unspecified |
| R06.03 | ICD-10 | Apnea |
| R06.09 | ICD-10 | Other abnormalities of breathing |
|  |  |  |

| **Table E3: Corticosteroids used in defining exacerbations** | | |
| --- | --- | --- |
| Brand Name | Generic Name |  |
| Prednisone | Prednisone |  |
| Deltasone | Prednisone |  |
| Prednicot | Prednisone |  |
| Sterapred | Prednisone |  |
| Rayos | Prednisone (delayed release) |  |
| Meticorten | Prednisone |  |
| DexPak 6 Day | Dexamethasone |  |
| DexPak 10 Day | Dexamethasone |  |
| DexPak 13 Day | Dexamethasone |  |
| LoCort | Hydrocortisone |  |
| ZonaCort | Hydrocortisone |  |
| Orapred ODT | Prednisolone (ODT) |  |
| Medrol | Methylprednisolone |  |
|  |  |  |

| **Table E4: IL21.7124.18.3 variants included in score** | | |
| --- | --- | --- |
| NPID_b38 | Effect | Effect_allele |
| 6:30698195:C:T | -0.025824749 | T |
| 6:30774965:A:C | 0.019060473 | A |
| 6:30801949:C:T | 0.017495621 | T |
| 6:30966017:G:T | 0.028502003 | T |
| 6:30967449:G:A | -0.011445445 | A |
| 6:30968237:T:C | -0.010272398 | T |
| 6:31011781:T:C | -0.001360813 | T |
| 6:31020736:G:A | 0.001675608 | A |
| 6:31025536:C:T | -0.006510993 | T |
| 6:31111122:G:T | -0.009363617 | T |
| 6:31137636:A:G | 0.015435378 | A |
| 6:31268690:G:T | 0.022458042 | T |
| 6:31272935:A:C | 0.005219471 | A |
| 6:31273862:A:G | -0.015185662 | A |
| 6:31281440:G:A | -0.01058551 | A |
| 6:31322081:A:G | -0.003851036 | A |
| 6:31329566:C:T | 0.00875427 | T |
| 6:31332509:A:G | -0.006998806 | A |
| 6:31336302:A:G | 0.000297473 | A |
| 6:31345256:G:A | -0.00264613 | A |
| 6:31345277:T:C | -0.003704404 | T |
| 6:31345825:A:C | 0.006957096 | A |
| 6:31347580:A:C | -0.00175774 | A |
| 6:31347929:G:A | 0.011499047 | A |
| 6:31351680:C:T | -0.035185425 | T |
| 6:31351956:G:A | -0.016260839 | A |
| 6:31352387:G:A | 0.003475852 | A |
| 6:31362841:C:T | -0.003443588 | T |
| 6:31363192:C:A | 0.010204419 | A |
| 6:31364750:G:A | -0.00703126 | A |
| 6:31365414:T:C | -0.00316413 | T |
| 6:31373136:A:G | 0.010856507 | A |
| 6:31379152:T:G | 0.008286302 | T |
| 6:31380263:T:C | -0.009459896 | T |
| 6:31383887:G:A | -0.007175466 | A |
| 6:31384263:T:C | -0.001008806 | T |
| 6:31462944:A:G | -0.010895258 | A |
| 6:31464810:T:C | -0.002206044 | T |
| 6:31469218:T:C | 0.017636957 | T |
| 6:31475005:G:A | -0.006101031 | A |
| 6:31479143:C:T | -0.01661859 | T |
| 6:31481706:G:A | 0.013815368 | A |
| 6:31493836:T:C | -0.025712715 | T |
| 6:31497884:T:G | -0.0226385 | T |
| 6:31504943:G:T | -0.006091615 | T |
| 6:31505470:C:T | -0.003263592 | T |
| 6:31507279:G:A | -0.007869942 | A |
| 6:31515359:A:G | -0.007275883 | A |
| 6:31519677:G:A | -0.015753298 | A |
| 6:31629923:C:T | -0.0424731 | T |
| 6:31629976:G:A | -0.028867799 | A |
| 6:31645962:T:C | -0.00077265 | T |
| 6:31772677:G:A | 0.014926084 | A |
| 6:31777507:C:T | 0.01131787 | T |
| 6:31812817:C:A | -0.03106069 | A |
| 6:31831299:T:G | 0.015853581 | T |
| 6:31858928:G:A | -0.00517996 | A |
| 6:31879859:G:A | -0.027934718 | A |
| 6:31913532:T:C | 0.020856832 | T |
| 6:31948623:A:G | -0.017724479 | A |
| 6:31961022:T:C | -0.007566002 | T |
| 6:31962574:A:G | 0.014201069 | A |
| 6:31962685:G:A | 0.010095511 | A |
| 6:31963360:T:C | -0.025303301 | T |
| 6:32030132:C:A | 0.008816385 | A |
| 6:32039847:A:G | -0.013987307 | A |
| 6:32043581:A:G | -0.044693129 | A |
| 6:32043937:A:G | -0.036502266 | A |
| 6:32049328:A:G | 0.020931017 | A |
| 6:32049465:C:T | 0.012405012 | T |
| 6:32050796:A:C | -0.011199718 | A |
| 6:32061638:T:C | -0.00278788 | T |
| 6:32064644:C:T | 0.017230927 | T |
| 6:32115523:A:G | -0.020529769 | A |
| 6:32121365:G:T | 0.022006845 | T |
| 6:32133100:C:T | 0.007415917 | T |
| 6:32138237:C:A | -0.00448251 | A |
| 6:32140074:G:A | -0.01432079 | A |
| 6:32142737:A:G | 0.036246168 | A |
| 6:32185629:C:A | -0.007112625 | A |
| 6:32196810:C:T | -0.012397367 | T |
| 6:32197667:A:G | 0.004432267 | A |
| 6:32208735:G:A | -0.015593261 | A |
| 6:32211932:A:G | 0.04013848 | A |
| 6:32224659:A:G | 0.027484316 | A |
| 6:32224840:T:C | 0.018592282 | T |
| 6:32226531:C:T | -0.022151879 | T |
| 6:32231367:C:A | -0.007150968 | A |
| 6:32235829:C:T | 0.000652811 | T |
| 6:32238466:C:T | 3.06E-05 | T |
| 6:32238842:G:T | -0.015984117 | T |
| 6:32240920:T:C | -0.012342886 | T |
| 6:32242166:T:C | 0.005119274 | T |
| 6:32243308:T:G | -0.007921498 | T |
| 6:32301176:G:A | -0.007628973 | A |
| 6:32316331:T:G | 0.010958285 | T |
| 6:32343682:G:A | -0.045982142 | A |
| 6:32345882:G:A | -0.006160539 | A |
| 6:32367236:G:A | 0.003346619 | A |
| 6:32372399:C:T | 0.007650053 | T |
| 6:32374914:C:A | 0.005180255 | A |
| 6:32387734:T:C | 0.02110985 | T |
| 6:32405921:G:T | -0.001178636 | T |
| 6:32407647:G:A | -0.004430586 | A |
| 6:32412413:G:A | 0.010345203 | A |
| 6:32413684:C:T | -0.020922263 | T |
| 6:32414162:T:C | -0.001819059 | T |
| 6:32420770:G:A | -0.005548422 | A |
| 6:32421768:A:G | 0.011907647 | A |
| 6:32421871:A:G | 0.01493041 | A |
| 6:32438565:A:C | 0.007828718 | A |
| 6:32440267:A:C | -0.027834881 | A |
| 6:32453692:G:A | -0.013921037 | A |
| 6:32454414:G:A | -0.009290527 | A |
| 6:32457620:C:A | -0.000754916 | A |
| 6:32457710:G:T | -0.003996708 | T |
| 6:32458206:T:C | -0.002717289 | T |
| 6:32465607:G:A | 0.037899052 | A |
| 6:32465869:C:T | 0.005211261 | T |
| 6:32472088:A:G | -0.005202018 | A |
| 6:32593839:T:C | -0.001997889 | T |
| 6:32598725:G:A | 0.016842635 | A |
| 6:32603378:T:C | -0.007049246 | T |
| 6:32604336:G:A | 0.005005428 | A |
| 6:32606583:C:T | 0.016668674 | T |
| 6:32610081:G:A | -0.009908556 | A |
| 6:32615478:C:T | -0.003646575 | T |
| 6:32615965:G:A | -0.005260708 | A |
| 6:32619010:A:G | 0.010204269 | A |
| 6:32620069:C:T | 0.00170397 | T |
| 6:32620680:T:C | 0.028499679 | T |
| 6:32623555:T:G | -0.002814507 | T |
| 6:32643917:G:A | -0.006652129 | A |
| 6:32646260:C:T | -0.014203813 | T |
| 6:32658309:G:A | 0.00917445 | A |
| 6:32680379:G:A | 0.010206818 | A |
| 6:32686220:A:G | 0.011691982 | A |
| 6:32689504:G:T | 0.00752028 | T |
| 6:32690847:G:A | -0.011763984 | A |
| 6:32693274:C:A | -0.001308899 | A |
| 6:32704576:A:G | 0.010251754 | A |
| 6:32706117:C:T | 0.014872238 | T |
| 6:32706948:A:G | -0.002295816 | A |
| 6:32710135:C:T | -0.00177951 | T |
| 6:32712755:T:C | 0.003189169 | T |
| 6:32714358:C:T | -0.010527856 | T |
| 6:32714617:T:G | 0.010066369 | T |
| 6:32714666:G:A | 0.004437117 | A |
| 6:32779725:A:G | -0.002344687 | A |
| 6:32832068:T:G | -0.014010342 | T |
| 6:32838896:T:C | -0.008794702 | T |
| 6:32911694:A:G | -0.006533411 | A |
| 12:7049176:T:C | -0.029027448 | T |
| 12:7052283:A:G | 0.027838066 | A |
| 12:7055497:C:T | 0.063062841 | T |
| 12:7064203:A:G | 0.088318163 | A |
| 12:7071568:T:C | 0.06012185 | T |
|  |  |  |

| **Table E5: IL5RA.4491.4.2 variants included in score** | | | |
| --- | --- | --- | --- |
| NPID_b38 | Effect | Effect_allele |  |
| 3:3066558:T:G | -0.168740286 | T |  |
| 3:3093484:G:A | 0.042702493 | A |  |
| 3:3094796:T:C | 0.067559666 | T |  |
| 3:3098273:T:C | -0.246964544 | T |  |
| 3:3099965:C:A | -0.103570388 | A |  |
| 3:3100109:C:T | -0.019990943 | T |  |
| 3:3100489:A:G | -0.071417578 | A |  |
| 3:3100819:C:A | -0.041014002 | A |  |
| 3:3102835:A:G | 0.171440458 | A |  |
| 3:3103081:C:T | 0.101617933 | T |  |
| 3:3103225:G:A | 0.103366274 | A |  |
| 3:3103843:A:G | 0.071709379 | A |  |
| 3:3105512:A:G | 0.033453632 | A |  |
| 3:3108029:A:C | 0.200472114 | A |  |
| 3:3109280:A:G | -0.079388003 | A |  |
| 3:3109662:A:G | -0.079187948 | A |  |
| 3:3110700:A:G | 0.240638082 | A |  |
| 3:3110805:G:A | 0.027917285 | A |  |
| 3:3111510:G:A | -0.091537162 | A |  |
| 3:3111655:G:A | 0.043193886 | A |  |
| 3:3112153:C:T | 0.037343166 | T |  |
| 3:3112912:G:A | -0.093292541 | A |  |
| 3:3113363:T:G | 0.103952263 | T |  |
| 3:3113545:G:A | -0.054556272 | A |  |
| 3:3113981:T:C | 0.002038221 | T |  |
| 3:3114457:G:A | 0.134600249 | A |  |
| 3:3117289:G:A | -0.023618411 | A |  |
| 3:3118329:C:T | 0.034105432 | T |  |
| 3:3118870:C:T | 0.062226934 | T |  |

|  |  | |  | |  |
| --- | --- | --- | --- | --- | --- |
| **Table E6: CCL17.3519.3.2 variants included in score** | | | | | |
| NPID_b38 | | Effect | | Effect_allele | |
| 3:16732951:T:C | | 0.177845073 | | T | |
| 3:16895741:A:G | | 0.256139298 | | A | |
| 3:42501269:C:T | | 0.044359953 | | T | |
| 3:42667981:C:T | | 0.012098309 | | T | |
| 3:42670027:C:T | | 0.053687779 | | T | |
| 3:42747590:G:A | | 0.059658445 | | A | |
| 3:42768995:G:A | | 0.082435663 | | A | |
| 3:42811954:A:G | | -0.032333698 | | A | |
| 3:42822312:A:G | | 0.015879404 | | A | |
| 3:42825467:A:G | | 0.005747074 | | A | |
| 3:42836219:A:G | | 0.006454286 | | A | |
| 3:42842084:C:T | | 0.001238808 | | T | |
| 3:42864624:T:C | | -0.232492788 | | T | |
| 3:42864724:C:T | | 0.161865365 | | T | |
| 3:42868195:A:G | | -0.020417725 | | A | |
| 3:42882284:G:T | | -0.011658172 | | T | |
| 3:42883199:G:A | | 0.081124881 | | A | |
| 3:42883463:T:C | | 0.09668799 | | T | |
| 3:42979009:T:C | | -0.027181776 | | T | |
| 3:43057303:C:T | | 0.009797756 | | T | |
| 8:105524455:T:G | | 0.054276451 | | T | |
| 8:105566712:T:C | | 0.084190127 | | T | |
| 8:105580979:C:A | | -0.091281276 | | A | |
| 10:119250744:A:G | | 0.145939441 | | A | |
| 10:119277642:C:T | | -0.077329071 | | T | |
| 16:57379053:A:C | | 0.095975784 | | A | |
| 16:57379218:C:T | | 0.040413625 | | T | |
| 16:57380089:G:A | | 0.047567763 | | A | |
| 16:57383222:G:T | | -0.067399328 | | T | |
| 16:57385075:G:A | | -0.032205167 | | A | |
| 16:57387107:G:A | | 0.096207975 | | A | |
| 16:57394562:C:T | | 0.006411056 | | T | |
| 16:57394953:A:G | | 0.054159248 | | A | |
| 16:57395387:G:A | | 0.002048647 | | A | |
| 16:57397022:T:G | | -0.126894081 | | T | |
| 16:57406984:T:C | | -0.025285455 | | T | |
| 16:57408279:T:C | | -0.174534501 | | T | |
| 16:57410651:G:T | | -0.012450463 | | T | |
| 16:57436972:A:C | | 0.118671355 | | A | |
| 16:57443740:C:T | | 0.026743143 | | T | |
| 16:57477157:A:G | | 0.019475072 | | A | |
| 16:57524596:T:G | | 0.122925087 | | T | |
|  | |  | |  | |

| **Table E7: DeLong P-values for comparing AUROCs** | | |
| --- | --- | --- |
|  | P-value | |
|  | MGB | AoU |
| **IL21** |  |  |
| Omalizumab | 0.60 | 0.82 |
| Dupilumab | 0.30 | 0.19 |
| Mepolizumab | 0.09 | 0.94 |
|  |  |  |
| ***IL21* vs. *IL21* + *IL5RA*** |  |  |
| Omalizumab | 0.33 | 0.76 |
|  |  |  |
| ***IL21* vs. *IL21* + *CCL17*** |  |  |
| Dupilumab | 0.87 | 0.36 |
|  |  |  |

| **Table E8: Evaluation of model calibration in each cohort with the Hosmer-Lemeshow test** | | |
| --- | --- | --- |
|  | P-value | |
|  | MGB | AoU |
| ***IL21*** |  |  |
| Omalizumab | 0.60 | 0.82 |
| Dupilumab | 0.30 | 0.19 |
| Mepolizumab | 0.09 | 0.94 |
|  |  |  |
| ***IL21* + *IL5RA*** |  |  |
| Omalizumab | 0.10 | 0.94 |
|  |  |  |
| ***IL21* + *CCL17*** |  |  |
| Dupilumab | 0.64 | 0.61 |
|  |  |  |

| **Table E9: Confusion matrices of predicted vs. observed response across models in MGBB and AoU cohorts.** | | | | | | | | |
| --- | --- | --- | --- | --- | --- | --- | --- | --- |
| **Model** | **True Positive** | **False Positive** | **True Negative** | **False Negative** | **Accuracy** | **Sensitivity** | **Specificity** | **Precision (PPV)** |
| Omalizumab |  |  |  |  |  |  |  |  |
| *IL21* in MGBB | 21 | 17 | 27 | 21 | 0.56 | 0.50 | 0.61 | 0.55 |
| *IL21* in AoU | 13 | 7 | 64 | 24 | 0.71 | 0.35 | 0.90 | 0.65 |
| *IL21*+*IL5RA* in MGBB | 22 | 20 | 24 | 20 | 0.54 | 0.52 | 0.55 | 0.52 |
| *IL21*+*IL5RA* in AoU | 13 | 9 | 62 | 24 | 0.69 | 0.35 | 0.87 | 0.59 |
|  |  |  |  |  |  |  |  |  |
| Dupilumab |  |  |  |  |  |  |  |  |
| *IL21* in MGBB | 27 | 7 | 4 | 3 | 0.76 | 0.90 | 0.36 | 0.79 |
| *IL21* in AoU | 31 | 15 | 18 | 10 | 0.66 | 0.76 | 0.55 | 0.67 |
| I*L21*+*CCL17* in MGBB | 28 | 7 | 4 | 2 | 0.78 | 0.93 | 0.36 | 0.80 |
| *IL21*+*CCL17* in AoU | 30 | 14 | 19 | 11 | 0.66 | 0.73 | 0.58 | 0.68 |
|  |  |  |  |  |  |  |  |  |
| Mepolizumab |  |  |  |  |  |  |  |  |
| *IL21* in MGBB IL5 | 9 | 6 | 14 | 7 | 0.64 | 0.56 | 0.70 | 0.60 |
| *IL21* in AoU IL5 | 0 | 0 | 47 | 10 | 0.83 | 0.00 | 1.00 | - |
|  |  |  |  |  |  |  |  |  |

**SUPPLEMENTARY TEXT**

The original score files for each GRS and SNP used in this study were derived from the larger UK Biobank–based cohort and are publicly available at omicspred.org.^1^ We have included the score files for the top variants: IL21.7124.18.3, IL5RA.4491.4.2, and CCL17.3519.3.2. Each file includes the variant identifier, chromosome and position, effect allele, and weight. The original score files were in genome build hg37, whereas both MGBB and All of Us genotyping data were in build hg38. Accordingly, the score files used here were lifted over to hg38.^2^ Scores were calculated using the plink –score function, which handles allele dosage, directionality, and strandedness. Because the GRSs varied widely in the number of contributing variants, we rank-normalized the scores to facilitate statistical analyses, rather than reporting effects per an arbitrary number of alleles. Trimodal distributions suggest additive genetic effects driven by a small number of variants and should not be treated as normally distributed variables, unlike GRSs. Therefore, for GRSs with trimodal distributions, we used the SNP with the largest effect size in the score file to represent the score and modeled it as a categorical variable. Principal components of genetic ancestry were calculated in the respective biobanks and methods have previously been described.

References

1. Sun BB, Maranville JC, Peters JE, et al. Genomic atlas of the human plasma proteome. Nature. Jun 2018;558(7708):73-79. doi:10.1038/s41586-018-0175-2
2. Hinrichs AS, Karolchik D, Baertsch R, et al. The UCSC Genome Browser Database: update 2006. Nucleic Acids Res. 2006 Jan 1;34(Database issue): D590-8. doi: 10.1093/nar/gkj144. PMID: 16381938.
